# Protective effects from Houttuynia cordata aqueous extract against acetaminophen-induced liver injury

**DOI:** 10.7603/s40681-014-0005-2

**Published:** 2014-08-27

**Authors:** Wei-ting Chen, Chieh-ling Yang, Mei-chin Yin

**Affiliations:** Department of Nutrition, China Medical University, 91, Hsueh-shih Rd, Taichung, Taiwan

**Keywords:** Hepatotoxicity, *Houttuynia cordata*, Acetaminophen, MCP-1, CYP2E1

## Abstract

**Background:**

Protective effects of *Houttuynia cordata* aqueous extract (HCAE) against acetaminophen-induced hepatotoxicity in Balb/cA mice were examined.

**Methods:**

HCAE, at 1 or 2 g/L, was added into the drinking water for 4 weeks. Acute liver injury was induced by acetaminophen treatment intraperitoneally (350 mg/kg body weight).

**Results:**

Acetaminophen treatment significantly depleted hepatic glutathione (GSH) content, increased hepatic malonyldialdehyde (MDA), reactive oxygen species (ROS) and oxidized glutathione (GSSG) levels, and decreased hepatic activity of glutathione peroxidase (GPX), catalase and superoxide dismutase (SOD) (*p*<0.05). The pre-intake of HCAE alleviated acetaminophen-induced oxidative stress by retaining GSH content, decreasing MDA, ROS and GSSG production, and maintaining activity of GPX, catalase and SOD in liver (*p*<0.05). The pre-intake of HCAE also significantly lowered acetaminophen-induced increase in cytochrome P450 2E1 activity (*p*<0.05). Acetaminophen treatment increased hepatic release of interleukin (IL)-6, IL-10, tumor necrosis factor (TNF)-alpha and monocyte chemoattractant protein-1 (*p*<0.05). HCAE intake significantly diminished acetaminophen-induced elevation of these cytokines (*p*<0.05).

**Conclusion:**

These results support that HCAE could provide hepato-protection

## 1. Introduction

Acetaminophen is an antipyretic and analgesic drug, and metabolized by cytochrome P450 system, which leads to the formation of. n-acetyl-pbenzoquinoneimine (NAPQI) [1,2] A large dose of this drug causes depletion of cellular glutathione (GSH) in liver because NAPQI reacts rapidly with GSH, which consequently enhances oxidation stress in conjunction with mitochondrial dysfunction, and leads to massive hepatocyte necrosis, liver failure or death [3,4]. On the other hand, it is known that cytochrome P450 2E1 (CYP2E1) regulates the metabolic activation of this drug both in human and rodents [5]. The increased CYP2E1 activity not only promotes NAPQI formation but also causes excessive reactive electrophiles and free radicals such as reactive oxygen species (ROS) to augment oxidative stress [6,7]. Thus, the agent(s) with GSH reserving ability and/or inhibitory effect upon CYP2E1 activity may provide preventive and/or alleviative effects for liver against the progression of acetaminophen-induced hepatocellular oxidative injury.

It is reported that interleukin-6 (IL-6) and tumor necrosis factor (TNF)-alpha are involved in acetaminophen-induced hepatotoxicity [8,9]. The increased release of these inflammatory cytokines, partially from the stimulation of oxidative stress [10], could consequently cause cytokine imbalance, immune dysfunction and even liver cell apoptosis. MCP-1 is generated at the site of infection or injury, and acts as a chemokine for monocyte recruitment and lymphocytes activation [11]. The increased hepatic mRNA expression of this chemokine has been observed in acetaminophen-treated mice, which facilitates the inflammatory response of liver innate immune system [12]. Thus, any agent(s) with suppressive effects on these inflammatory cytokines and/or chemokines may be able to improve acetaminophen-induced liver injury.

*Houttuynia cordata* is traditionally used as a medicinal plant in Asia countries including China, Taiwan, Japan and Thai [13]. It is reported that *Houttuynia cordata* provided anti-oxidative protection in mice against frying-oil and CCl4-induced injury [14,15]. Our previous study found that *Houttuynia cordata* aqueous extract (HCAE) was rich in phenolic acids and flavonoids; and HCAE intake at 1 and 2% suppressed high fat diet induced oxidative and inflammatory stress in heart and liver via reducing malondialdehyde level, retaining GSH content and glutathione peroxidase activity, declining TNF-alpha, IL-1beta and IL-6 production [16]. Those previous studies suggest that HCAE may provide nutritional benefit for liver. However, it remains unknown that HCAE could protect liver against acetaminophen-induced oxidative and inflammatory damage.

The purpose of this animal study was to examine the protective effects of *Houttuynia cordata* aqueous extract on liver of acetaminophen treated mice. The influence of this extract upon CYP2E1 activity, associated antioxidant enzymes activities, and cytokines were also evaluated.

## 2. Materials and Methods

### 2.1 Materials

Fresh *Houttuynia cordata* leaves, harvested in summer, 2012, were obtained from Nantou County, Taiwan. *Houttuynia cordata* aqueous extract (HCAE) was prepared by mixing 100 g chopped leaves and 250 mL sterile distilled water, homogenizing in a Waring blender and cooking for 20 min at 100°C. After filtrating through a Whatman No. 1 filter paper, the filtrate was further freeze-dried to a fine powder. Our previous study indicated that HCAE had total phenolic acids at 2175±210 mg/100 g dry HCAE. In our present study, the content of total phenolic Corresponding author, Department of Nutrition, China Medical University, 91, Hsueh-shih Rd., Taichung City, Taiwan

### 2.2. Animals and diets

Four- to five-week-old male Balb/cA mice were obtained from National Laboratory Animal Center (National Science Council, Taipei City, Taiwan). Mice were housed on a 12-h light-12-h dark schedule, and fed with water and mouse standard diet (PMI Nutrition International LLC, Brentwood, MO, USA). Use of the mice was reviewed and approved by the China Medical University animal care committee.

### 2.3. Experimental design

HCAE at 1 or 2 g/L was directly added into the drinking water. Two control groups of mice consumed distilled water, and all mice consumed normal diet. After 4 weeks supplement, HCAE treated mice and one control group were treated by APAP intraperitoneally (ip 350 mg/kg body weight), and all mice were sacrificed after 24 h. Liver from each mouse was collected and weighted. Blood was also collected, and plasma or serum was separated from erythrocyte immediately. Liver tissue, 100 mg, was homogenized on ice in 1 mL phosphate buffer (pH 7.2), and the filtrate was collected. Protein concentration of tissue homogenate was determined by a commercial assay kit (Pierce Biotechnology Inc., Rockford, IL, USA) with bovine serum albumin as standard. In all experiments, sample was diluted to a final concentration of 1 g protein /L.

### 2.4. Alanine aminotransferase (ALT), aspart ate aminotransferase (AST) and c-reactive protein (CRP) analyses

Serum activities of ALT and AST were determined by using commercial assay kits (Randox Laboratories Ltd., Crumlin, UK). CRP level (mg/L) was determined by a commercial ELISA kit (Anogen, ON, Canada).

### 2.5. GSH and oxidized glutathione (GSSG) levels, superoxide dismutase (SOD), catalase and glutathione peroxidase (GPX) activities assay

GSH and GSSG concentrations (nmol/mg protein) in liver were determined by commercial colorimetric GSH and GSSG assay kits (OxisResearch, Portland, OR, USA). Catalase, SOD and GPX activities (U/mg protein) in liver were determined by catalase, SOD and GPX assay kits (Calbiochem, Inc., San Diego, CA, USA)

### 2.6. Determination of lipid oxidation and ROS

Lipid oxidation in liver was determined by measuring the level of malondialdehyde (MDA, μmol/L) via an HPLC method [6]. The method described in Gupta et al. [17] was used to measure hepatic ROS level. Briefly, 10 mg liver was homogenized in 1 mL of ice cold 40 mM Tris–HCl buffer (pH 7.4), and further diluted to 0.25% with the same buffer. Then, samples were divided into two equal fractions. In one fraction, 40 μL 1.25 mM 2’,7’-dichlorofluorescin diacetate in methanol was added for ROS estimation. Another fraction, in which 40 μL methanol was added, served as a control for auto fluorescence.

After incubating for 15 min at 37 °C, fluorescence was determined at 488 nm excitation and 525 nm emission using a fluorescence plate reader.

### 2.7. Cytokines measurements

Hepatic levels of IL-6, IL-10, TNF-alpha and MCP-1 were measured by using cytoscreen immunoassay kits (BioSource International, Camarillo, CA, USA). Samples were run in duplicates according to manufacturer’s instructions

### 2.8. Measurement of CYP2E1 activity

The activity of CYP2E1 in liver microsome was estimated by colorimetrically measuring the formation of 4-nitrocatechol, a product from p-nitrophenol hydroxylation catalyzed specifically by CYP2E1. The protein concentration of CYP2E1 was measured by ELISA, and a rabbit anti-CYP2E1 antibody (Calbiochem, Inc., San Diego, CA, USA) was used as the detect system. The formed 4-nitrocatechol was expressed as nmol/mg protein.

### 2.9. Statistical analyses

The effect of each treatment was analyzed from 10 mice (n=10) in each group. Data were subjected to analysis of variance (ANOVA). Differences with *p*<0.05 were considered to be significant.

## 3. Results

**Fig. 1:**
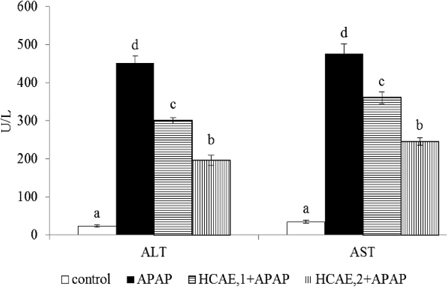

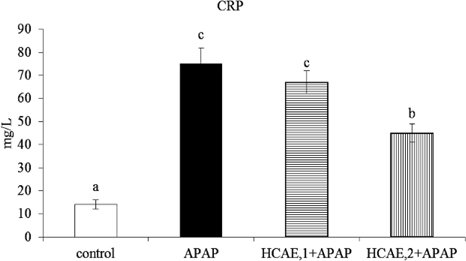
Plasma ALT, AST and CRP levels in mice pre-treated by 0, 1 or 2 g/L HCAE, and followed with acetaminophen (APAP) treatment. Control group was mice without HCAE intake and without APAP treatment. Data are mean ± SD (n=10). ^a-d^Means among bars without a common letter differ, *p*<0.05.

As shown in Table 1, the intake of HCAE did not affect daily water intake, feed intake, final body weight and liver weight (*P*>0.05). Plasma levels of ALT, AST and CRP are presented in Figure 1. The pre-treatments of HCAE alleviated acetaminophen-induced elevation of ALT and AST (*p*<0.05). HCAE intake only at 2 g/L significantly declined acetaminophen-induced increase in CRP (*p*<0.05).

**Table:** 

	water	HCAE, 1	HCAE, 2
WI	2.7±0.2	2.9±0.3	2.6±0.3
FI	2.1±0.3	2.2±0.2	2.4±0.4
BW	25.3±1.2	24.8±1.0	25.0±0.8
LW	1.29±0.11	1.35±0.14	1.24±0.12

**Fig. 2:**
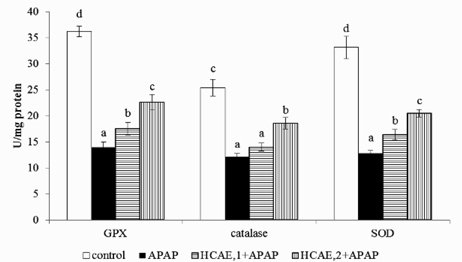
Hepatic GPX, catalase and SOD activities in mice pre-treated by 0, 1 or 2 g/L HCAE, and followed with acetaminophen (APAP) treatment. Control group was mice without HCAE intake and without APAP treatment. Data are mean ± SD (n=10). ^a-d^Means among bars without a common letter differ, *p*<0.05.

**Fig. 3:**
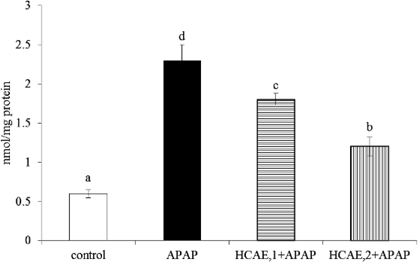
Liver microsomal CYP2E1 activity, determined as 4-nitrocatechol (nmol/mg protein), from mice pre-treated by 0, 1 or 2 g/L HCAE, and followed with acetaminophen (APAP) treatment. Control group was mice without HCAE intake and without APAP treatment. Data are mean ± SD (n=10). ^a-d^Means among bars without a common letter differ, *p*<0.05.

Acetaminophen treatment significantly decreased hepatic GSH, and increased GSSG, MDA and ROS levels (Table 2, *p*<0.05); but the pre-intake of HCAE alleviated acetaminophen-induced GSH depletion, and reduced GSSG, MDA and ROS formation (*p*<0.05). As shown in Figure 2, acetaminophen treatment significantly reduced hepatic GPX, catalase and SOD activities (*p*<0.05). However, the pre-intake of HCAE significantly retained GPX and SOD activities (*p*<0.05). HCAE intake only at 2 g/L significantly maintained hepatic catalase activity (*p*<0.05). Acetaminophen treatment raised CYP2E1 activity (Figure 3), and HCAE pre-intake significantly suppressed subsequent acetaminophen-induced elevation of CYP2E1 activity (*p*<0.05). As shown in Table 3, acetaminophen treatment also significantly increased hepatic levels of TNF-alpha, IL-6, IL-10 and MCP-1 (*p*<0.05). The pre-intake of HCAE decreased acetaminophen-induced release of IL-6, IL-10 and MCP-1 (*p*<0.05). HCAE pre-intake only at 2 g/L significantly lowered hepatic TNF-alpha level (*p*<0.05).

**Table 2:** Hepatic content of GSH (nmol/mg protein), GSSG (nmol/mg protein), MDA (μmol/L) and ROS (nmol/mg protein) from mice pre-treated by 0, 1 or 2 g/L HCAE, and followed with acetaminophen (APAP) treatment. Control group was mice without HCAE intake and without APAP treatment. Data are mean ± SD (n=10).

	control	APAP	HCAE,1+APAP	HCAE,2+APAP
GSH	22.7±1.0^d^	12.1±0.5^a^	15.9±0.6^b^	18.0±0.8^c^
GSSG	0.26±0.05^a^	1.22±0.08^d^	0.96±0.10^c^	0.65±0.06^b^
MDA	0.51±0.07^a^	1.58±0.10^d^	1.21±0.09^c^	0.88±0.05^b^
ROS	0.34±0.03^a^	1.64±0.12^d^	1.25±0.06^c^	0.79±0.08^b^

^a-d^Means in a column without a common letter differ, *p*<0.05.

**Table 3:** Hepatic level (pg/mg protein) of TNF-alpha, IL-6, IL-10 and MCP-1 from mice pre-treated by 0, 1 or 2 g/L HCAE, and followed with acetaminophen (APAP) treatment. Control group was mice without HCAE intake and without APAP treatment. Data are mean ± SD (n=10).

	control	APAP	HCAE,1+APAP	HCAE,2+APAP
TNF-alpha	17±4^a^	523±21^c^	495±14^c^	407±17^b^
IL-6	16±2^a^	419±18^d^	366±10^c^	290±12^b^
IL-10	15±5^a^	303±15^d^	232±13	157±9^b^
MCP-1	14±3^a^	568±25^d^	475±18^c^	369±11^b^

^a-d^Means in a column without a common letter differ, *p*<0.05.

## 4. Discussion

Our previous study found that HCAE was rich in phenolic acids and flavonoids, and the intake of HCAE at 1 or 2% lowered hepatic and circulating lipid accumulation, as well as attenuated oxidative and inflammatory stress in liver from mice consumed high fat diet [16]. Our present study further found that the pre-intake of HCAE markedly protected liver against subsequent acetaminophen-induced oxidative and inflammatory injury via decreasing ALT and AST levels, increasing GSH retention, suppressing CYP2E1 activity, lowering IL-6 and MCP-1 release. These results support that HCAE is a potent hepatoprotective agent.

It is well known that acetaminophen at high dose causes GSH depletion, ROS accumulation and inflammatory cytokines release in liver [18,19]. The results from our present study agreed those previous studies. Furthermore, we found that the pre-intake of HCAE effectively alleviated acetaminophen-induced GSH depletion and GSSG increase in liver. It seems that hepatoprotective effects from HCAE could be partially ascribed to HCAE maintain hepatic GSH content. We also found that the pre-intake of HCAE retained the activity of three antioxidant enzymes in liver of acetaminophen-treated mice. Obviously, HCAE could provide hepatic anti-oxidative protection via enzymatic actions. CYP2E1 is the major isozyme responsible for the formation of NAPQI from acetaminophen [20,21]. A high dose of acetaminophen elevates CYP2E1 activity and facilitates this catalytic reaction, which in turn promotes ROS overproduction. In our present study, HCAE pre-intake markedly declined subsequent acetaminophen-induced CYP2E1 activity elevation, lowered ROS formation, and reduced acetaminophen-induced oxidative stress. These results implied that the protective action from HCAE was partially due to its suppressive effect on CYP2E1 activity.

IL-6 and TNF-alpha, proinflammatory cytokines, were central mediators for the regulation of several biomarkers such as CRP, especially at acute phase response [22]. In our present study, acetaminophen treatment increased the release of these proinflammatory cytokines and CRP; however, the pre-intake of HCAE decreased hepatic formation of these inflammatory cytokines and circulating CRP. Apparently, the anti-inflammatory protection from this extract was partially due to its inhibitory effects on the production of these cytokines. Dambach et al. [8] indicated that the increased IL-6 and TNF-alpha from acetaminophen treatment could induce neutrophil accumulation and activation at liver; stimulate macrophage and hepatocyte production of nitric oxide, which further enhance acetaminophen-induced hepatoxicity. MCP-1 is a chemotactic factor for activating monocytes and macrophages, and could recruit monocytes to the sites of injury [23,24]. In our present study, the increased hepatic MCP-1 and TNF-alpha levels indicated that the livers of these mice were injured, and implied that neutrophil and macrophage were activated to promote inflammatory reactions. However, the pre-intake of HCAE substantially decreased hepatic TNF-alpha and MCP-1 generation in acetaminophen-treated mice, which suggested that this extract might protect liver against inflammation via diminishing the activation of neutrophil, monocytes and macrophages, reducing the recruitment of monocytes. It is well known that oxidation and inflammation are closely interrelated in biological systems [25]. Since the pre-intake of HCAE already coped with acetaminophen-induced oxidative stress, it is reasonable to observe the lower levels of inflammatory cytokines in HCAE treated mice.

Our previous study reported that 8 phenolic acids and 7 flavonoids such as gallic acid, ellagic acid, ferulic acid, kaempferol, myricetin and quercetin could be detected in HCAE [16]. It is reported that these compounds possess anti-oxidative and/or anti-inflammatory activities [26,27]. Thus, the hepatic protection from this extract against acetaminophen as we observed could be due to the presence of these phenolic acids and flavonoids.

In conclusion, *Houttuynia cordata* aqueous extract may be considered as hepatoprotective agent because the pre-intake of this extract protected liver against subsequent acetaminophen-induced oxidative and inflammatory injury via retaining hepatic GSH content, maintaining GPX and SOD activities, suppressing CYP2E1 activity and decreasing production of IL-6 and MCP-1.

## Acknowledgements

This study was supported by a grant from China Medical University, Taichung City, Taiwan (CMU 102-AWARD-04).
